# Kv3.3b expression defines the shape of the complex spike in the Purkinje cell

**DOI:** 10.3389/fncel.2013.00205

**Published:** 2013-11-13

**Authors:** Ken Veys, Dirk Snyders, Erik De Schutter

**Affiliations:** ^1^Theoretical Neurobiology, University of AntwerpAntwerpen, Belgium; ^2^Lab for Molecular Biophysics, Physiology and Pharmacology, University of AntwerpAntwerpen, Belgium; ^3^Computational Neuroscience Unit, Okinawa Institute of Science and TechnologyOkinawa, Japan

**Keywords:** single cell PCR, Purkinje, cerebellum, RNA amplification, potassium channels, Kv3.3b, Real time PCR, complex spike

## Abstract

The complex spike (CS) in cerebellar Purkinje Cells (PC) is not an all-or-nothing phenomena as originally proposed, but shows variability depending on the spiking behavior of the Inferior Olive and intrinsic variability in the number and shape of spikelets. The potassium channel Kv3.3b, which has been proposed to undergo developmental changes during the postnatal PC maturation, has been shown to be crucial for the repolarization of the spikelets in the CS. We address here the regulation of the intrinsic CS variability by the expression of inactivating Kv3.3 channels in PCs by combining patch-clamp recordings and single-cell PCR methods on the same neurons, using a technique that we recently optimized to correlate single cell transcription levels with membrane ion channel electrophysiology. We show that while the inactivating TEA sensitive Kv3.3 current peak intensity increases with postnatal age, the channel density does not, arguing against postnatal developmental changes of Kv3.3b expression. Real time PCR of Kv3.3b showed a high variability from cell to cell, correlated with the Kv3.3 current density, and suggesting that there are no mechanisms regulating these currents beyond the mRNA pool. We show a significant correlation between normalized quantity of Kv3.3b mRNA and both the number of CS spikelets and their rate of voltage fluctuation, linking the intrinsic CS shape directly to the Kv3.3b mRNA pool. Comparing the observed cell-to-cell variance with studies on transcriptional noise suggests that fluctuations of the Kv3.3b mRNA pool are possibly not regulated but represent merely transcriptional noise, resulting in intrinsic variability of the CS.

## INTRODUCTION

The cerebellar Purkinje cell (PC) is a fast spiking inhibitory neuron and represents the only projection from the cerebellar cortex to the cerebellar nuclei (Ito, 2006). It receives two inputs: the parallel fiber input yielding small postsynaptic potentials whereas the climbing fiber input results in a massive depolarization of the dendrite. The latter gives rise to the complex spike (CS) in the soma, which is a large calcium current driven depolarization with one large spike followed by one to six smaller spikelets (Eccles et al., 1964; Eccles, 1967; Thach, 1967). The timing and number of spikelets is a reflection of the bursting of the inferior olive (Mathy et al., 2009) but also of inter-PC variability in CS shape (Zagha et al., 2008). Previous work showed that Kv3.3b channels are crucial for the repolarization of the spikelets (Zagha et al., 2008). Kv3.3b channels are presumed to be under developmental control and cause the narrowing of Na^+^-spikes and the emergence of a fast after hyperpolarization during postnatal development (McKay and Turner, 2004, 2005).

Purkinje cells express three TEA sensitive voltage-dependent potassium channels that belong to the Shal family: Kv3.1, Kv3.3b, and Kv3.4 (Weiser et al., 1994; Rudy and McBain, 2001) of which the homo-tetramer channels of Kv3.1 act as a true delayed rectifier with almost no inactivation (τ = 630 ms, Grissmer et al., 1992). Both Kv3.3b and Kv3.4 express as a transient or A-type current, but inactivation of Kv3.4 channels (τ = 15.9 ms, Rettig et al., 1992) is much faster than Kv3.3 (τ = 200 ms, Vega-Saenz de Miera et al., 1992). The subunits of the Kv3 family have the ability to form hetero-tetramers, which yield currents similar to the homo-tetrameric channel that make them. Kv3.2 is not expressed in the PC (Weiser et al., 1994; Martina et al., 2003). Several groups have shown using immunocytochemistry that the PC soma and dendrites stain for Kv3.3 (Martina et al., 2003; Joho and Hurlock, 2009). Staining for Kv3.4 is weak in the soma and proximal dendrites, but very strong for distal dendrites (Martina et al., 2003).

The goal of this study was to investigate if the Kv3.3 channels indeed show developmental regulation and whether such regulation affects their control over the repolarization of CS spikelets. Such regulatory mechanisms could exist on different levels: the size of the mRNA pool of Kv3.3b, regulated by transcription and degradation rates, and/or the rate of translation and post-translational modifications. Our research focused on the first by combining patch clamping with single cell mRNA measurements. We show that the Kv3.3b current density does not correlate with age but does correlate with the amount of Kv3.3b mRNA. Furthermore, we found that the shape of the CS is related to the Kv3.3b mRNA content of the PC.

## MATERIALS AND METHODS

### ETHICS STATEMENT

All procedures were performed in accordance with the European guidelines for the care and use of laboratory animals (86/609/EEC) and were approved by the Committee on Animal Care and Use at the University of Antwerp, Belgium. All efforts were made to reduce the number of animals and to minimize animal suffering. Mice were deeply anesthetized with isoflurane followed by decapitation.

### SLICE PREPARATION AND SOLUTIONS

Cerebellar brain tissue slices were obtained by standard methods from C5bl/6j mice ranging from 4 days postnatal to 23 days for measuring the kv3.3b current density in voltage clamp. CS experiments were done in current clamp using mice from 18 to 24 days old. All experiments were conducted in agreement with institutional guidelines on animal experimentation. Briefly, after quick decapitation the cerebellum was removed in ice-cold artificial cerebrospinal fluid (ACSF) containing (in millimoles) 62 NaCl, 3 mM MgCl_2_, 1 CaCl_2_, 2.5 KCl, 1.25 NaH_2_PO_4_, 26 NaHCO_3_, 10 glucose (Merck, Germany) adjusted to 320 mOsm by sucrose (Analar Normapure, Belgium). Sagital cerebellar slices from the vermis were cut by a VT 1000s vibratome (Leica, Zaventem, Belgium) in an oxygenated “reversed” ice cold solution resembling intracellular solution containing (in millimoles): 130 K-gluconate, 2.5 KCl, 2 EGTA, 20 HEPES, 25 glucose, whose pH was adjusted with NaOH. The slices were incubated at 32°C for only 5 min and left afterward at room temperature until transfer to the recording chamber to minimize mRNA degradation (Hongpaisan and Roomans, 1997). During incubation, storage, and during the initial steps of the electrophysiological recordings, slices were bathed and perfused in ASCF containing (in millimoles) 125 NaCl, 4 KCl, 2 MgSO_4_, 26 NaHCO_3_, 2 CaCl_2_, 25 glucose, constantly balanced with 95% O_2_ and 5% CO_2_.

Recordings were performed under constant perfusion at a rate of 2 ml/min of ACSF. Upon establishment of the whole-cell patch-clamp configuration (Hamill et al., 1981), the perfusion was switched to a calcium-free ACSF. Chemicals were obtained from Sigma, Germany, unless stated otherwise.

For voltage clamp experiments the pipette was filled with RNAse free solution containing (in millimoles): 40 KCl, 2 MgCl_2_, 20 HEPES, 5 EGTA, 5 K_4_BAPTA, 80 NMG, pH corrected to 7.3 with HCl. By using only 60 mM of K^+^ we reduced the Kv3.3 current amplitude, improving the voltage clamp recordings of this current.

For current clamp experiments the pipette was filled with RNAse free solution containing (in mM): 144 K Gluconate, 3 MgCl, 10 HEPES, 0.2 EGTA, 4 MgATP, 0.5 NaGTP pH corrected to 7.4 with KOH.

### ELECTROPHYSIOLOGY

The single-electrode voltage-clamp technique was performed by means of an EPC10 amplifier (Heka, Lambrecht/Pfalz, Germany), controlled by the Pulse software (Heka, Lambrecht/Pfalz, Germany). Pipettes were pulled with a P-97 micropipette puller (Sutter instruments, Novato, CA, USA) and had a resistance ranging from 2 to 3 MΩ.

Experiments were performed at room temperature (20–22°C). The recordings were low-pass filtered at 10 KHz and sampled at 25 kHz. Data were analyzed by custom routines written in Igor Pro 6.05A (WaveMetrics, Portland, OR, USA). Capacitance, membrane resistance, and series resistance were estimated by an AC-method, i.e., the Lindeau–Neher technique, by means of the lock-in extension of the EPC-10 amplifier (Gillis, 2000). About 50 and 500 Hz sinusoidal voltage command waveforms, with 10 mV amplitude and -100 mV offset were used. The low frequency of 50 Hz was chosen for optimally estimating the total membrane capacitance, regardless of the different stages of the cell development. The higher frequency of 500 Hz was chosen to estimate the membrane capacitance of the soma and proximal dendrites (Sacco and Tempia, 2002).

**FIGURE 1:**
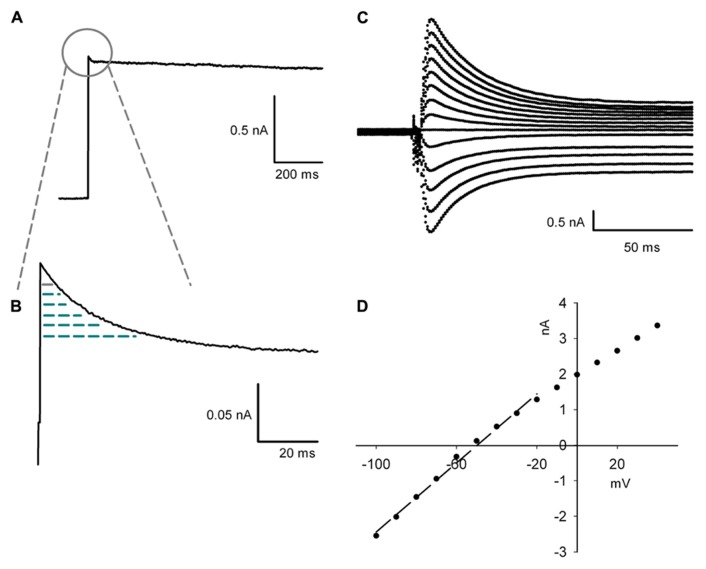
**Protocol for measuring the capacitance. (A)** The capacitance of the voltage clamped area is derived form the partial integration of the capacitive transient during a step from -50 to -20 mV with 3 mM of TEA blocking inactivating Kv3.3 currents. **(B)** Enlargement of the capacitive transient which is integrated from the start of the depolarization to the time point when the TEA sensitive inactivating current reaches its peak amplitude. The integrated transient is represented as the arced area. **(C)** Enlargement of capacitive transients evoked by 10 mV increment steps from -100 to +40 mV starting from a holding potential of -50 mV. **(D)**, I-V plot of the peak of the capacitive transient shown in **(C)** compared to the membrane potential. The arced line shows the linear regression between -100 and -20 mV (*R*^2^ = 0.99).

Due to the extensive remodeling of the dendritic tree the space-clamp error changes during maturation of PCs. Therefore, the electrophysiological measurement of conductance should not be normalized by the complete membrane surface or capacitance, but by the capacitance of the effectively clamped area (Taylor, 2012). This was inferred by integrating the capacitive transient evoked by stepping the voltage command from -50 to -20 mV under bath application of 3 mM TEA (blocking the transient Kv3.3b current) and 1 μM TTX (**Figures 1A,B**; Taylor, 2012). A depolarization to -20 mV was possible as the capacitive currents were linear between -100 and -20 mV, showing that there were no active currents using this protocol (**Figures 1C,D**). This transient was integrated form the start of the voltage step until the time of peak of the inactivating TEA sensitive potassium current. The integrated transient was employed as the capacitance of the clamped membrane area of the cell with the aim of removing the dependence of current density on the total membrane surface under space-clamp errors (**Figure 3D**). Series resistance compensation between 80 and 90% was used for current recordings (except when measuring the capacitance of the clamped area of the cell). For this the access resistance and capacitance of the soma were used as calculated online by the 500 Hz lockin experiments instead of the auto function of the HEKA amplifier. By this manipulation overloading of the soma was avoided (Major, 1993).

**FIGURE 2:**
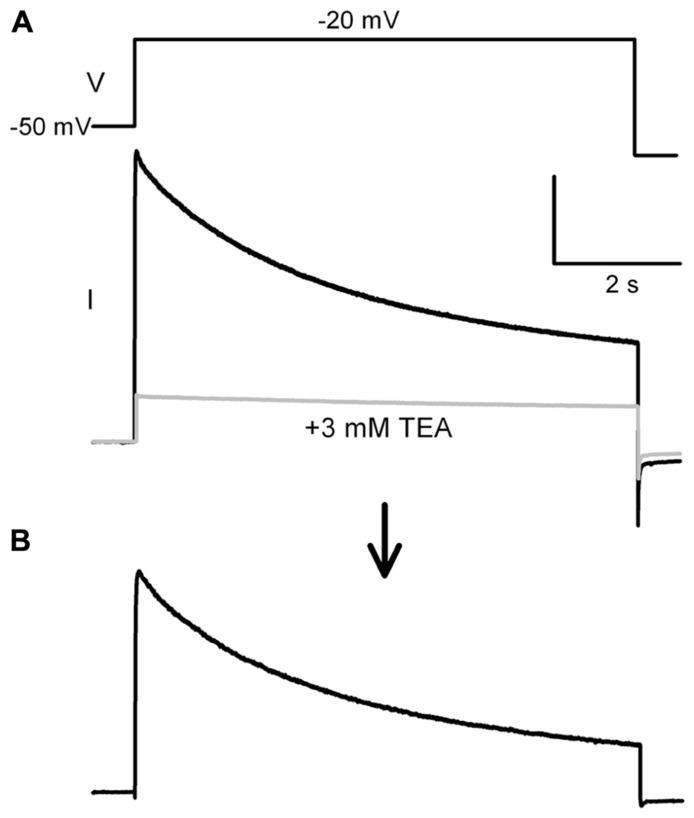
**Protocol for measuring TEA sensitive potassium current. (A)** The potassium current was measured with a step protocol from a –50 mV prepulse to -20 mV, as shown in the inset above the trace. This protocol was run a second time with 3 mM TEA (gray trace). **(B)** Offline subtraction yielded the TEA sensitive current. The TEA sensitive inactivating current was measured between the peak current and the steady state current end of the step.

Outward potassium currents were measured somatically under voltage-clamp and evoked by a voltage-step depolarization protocol. Throughout the recordings, cells were kept at a holding potential of -80 mV. During each stimulation, a 5-s prepulse to -50 mV was delivered in order to fully inactivate subthreshold A-type *I*_k_. The voltage-dependence of the remaining K-currents were revealed by 6-s pulses to potentials between -100 and +40 mV. The interstimulus interval was 8 s. The same protocol was repeated after bath application of 3 mM TEA. Individual traces recorded without and with TEA were subtracted offline. The inactivating Kv3.3 currents were measured by subtracting residual currents after the 6 s depolarization step ( **Figures 2A,B**). Both calcium currents and calcium-dependent potassium currents were abolished by switching to a calcium-free ACSF upon the establishment of the whole-cell configuration. Sodium currents were blocked with 1 μM TTX (Tocris, UK).

Complex spike waveforms were evoked by placing a theta glass electrode (Hilgenberg, Malsfeld, Germany), filled with ACSF, in the granule cell layer directly beneath the targeted PC. Single stimulations (0.2 ms, 200–2000 μA) delivered by an ISO-Flex stimulus isolator (A.M.P.I., Jerusalem, Israel) were used to activate climbing fibers as described previously (Zagha et al., 2008). Repetitive stimulations were conducted at 0.1 Hz to minimize potential synaptic plasticity (Hansel and Linden, 2000). The rate of voltage fluctuation of the spikelets was calculated as SD of the first derivative of the voltage trace during the 10 ms window after the first spike of the CS (**Figures 6A,B**).

### REAL TIME PCR ANALYSIS OF SINGLE PCs

The RNA content of a single PC was extracted, reverse transcribed, and amplified by a RNA amplification as previously described (Veys et al., 2012).

Real-Time PCR was performed on a Lightcycler 480 (Roche, Brussel, Belgium) using its probes master mix. The primers for calbindin 28K were: AATCCCACCTGCAGTCATCT (sense), CCAGGTAACCACTTCCGTCA (antisense), and TGAGATCTGGCTTCATTTCGACGC (probe) based on NM_009788. The primers for Glyceraldehyde 3-phosfate: TTCACCACCATGGAGAAGGC (sense), GGCATGGACTGTGGTCATGA (antisense), and TGCATCCTGCACCACCAACTGCTTAG (probe) based on NM_008084.2.

Both primers were custom designed and obtained from Eurogentec (Liège, Belgium). Kv3.3b primers was purchased from Applied Biosystem (Carlsbad, CA, USA) as a custom order based on S69381.1 (Goldman-Wohl et al., 1994).

Cycle threshold (Ct) levels were rescaled by the average for each gene and transformed into relative quantities using the amplification efficiency. (calbindin 28K and Kv3.3b : 1.97 and Glyceraldehyde 3-phosfate: 2). The normalized quantity of Kv3.3b was calculated by dividing the relative quantity by the averaged quantities of the two housekeeping genes (Vandesompele et al., 2002).

### STATISTICAL ANALYSIS

The coefficient of variation (Cv) of the normalized current density was calculated as the standard deviation divided by the mean. The normalized expression of KV3.3b was compensated for the age dependency by a linear fit (slope: 0.071) and the residual data was used to calculate the Cv after subtracting the standard deviation of the real time reaction, which was calculated as previously described (Pfaffl, 2001).

## RESULTS

Zagha et al. (2008) showed that the CS displays intrinsic inter-cell variability in its shape and number of spikelets. Repolarization of the spikelets has been attributed mostly to a somatic delayed rectifier channel, namely Kv3.3b (Zagha et al., 2008). It has not been investigated whether Kv3.3b currents are tightly regulated in PCs and if such a mechanism can influence the shape of the CS and the number of spikelets. The same somatic channel has previously been suggested to be under developmental control (Goldman-Wohl et al., 1994; McKay and Turner, 2004, 2005). If such a mechanism exists, it would also control the CS shape, and therefore has to be taken under consideration.

### ARE Kv3.3b CURRENTS UNDER DEVELOPMENTAL CONTROL?

Our pilot experiments with real-time PCR of PCs showed that Kv3.1 expression was weak: only one cell (p7) had expression above the threshold of detection for both replicates; eight cells had one positive result out of the two replicates and five cells were negative in both. This was consistent with a low expression of Kv3.1 in the PC (Weiser et al., 1994) that declines during maturation (Martina et al., 2003). We could not detect Kv3.4 in the PC somata.

All Kv3 channels are very sensitive to external tetraethylammonium (TEA; at concentrations less than 3 mM). PCs also express other delayed rectifiers like Kv1.1, Kv1.2, Kv1.3, and Kv1.6 (Veh et al., 1995), but these channels are not sensitive to TEA except for Kv1.1. However, it has been reported that currents of the Kv1 family are not inactivating in the PC (McKay et al., 2005), so they could be differentiated from Kv3 currents by a subtraction protocol (see Materials and Methods). We measured inactivating Kv3 currents using a low K^+^ internal solution to reduce total current amplitude. Kv3 currents were isolated by eliminating all sub threshold potassium currents with a -50 mV pre pulse and by subtracting non-TEA sensitive currents as non-inactivating currents which are not TEA-sensitive (**Figure 2**). There is almost no inactivation of Kv3.1 currents during the 6 s pulse (Grissmer et al., 1992), while Kv3.3b currents are inactivated by more than 80% (Vega-Saenz de Miera et al., 1992).

All currents were measured at -20 mV in order to reduce the error of series resistance.

We could not measure reliable kinetic data as the currents at +20 mV were too big (especially in older cells) and were therefore not trustworthy. However, preliminary data showed a half activation of -19 mV (data not shown) which was more negative than reported by Sacco et al. in p4–p7 mice (-5 mV; Sacco et al., 2006) but more positive than reported by McKay and Turner (2004).

**FIGURE 3:**
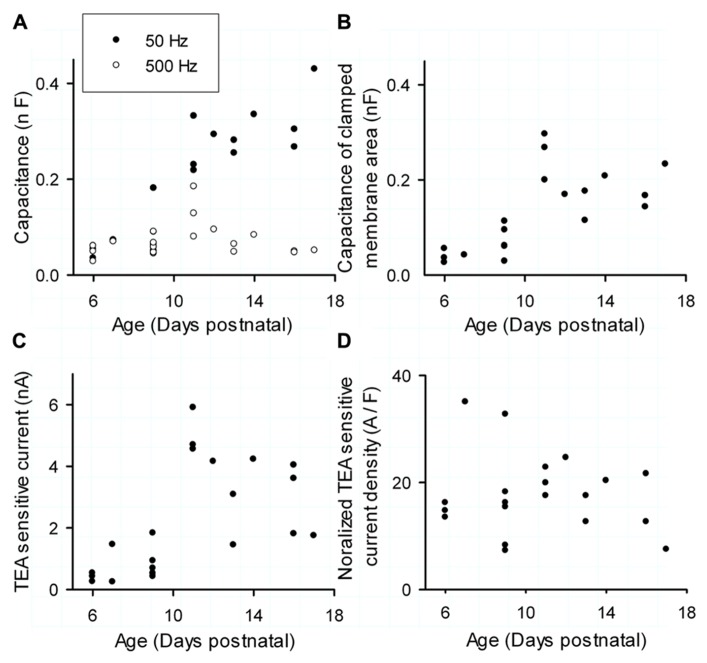
**Maturation of Kv3 expression during postnatal development. (A)** Total capacitance (filled circles, measured with a 50-Hz sinus wave) and capacitance of soma and proximal dendrite (circles, measured with a 500 Hz sinus wave) versus postnatal age. **(B)** Capacitance of the clamped membrane area of the cell versus postnatal age. **(C)** TEA- sensitive Kv3 current versus postnatal age. **(D)** Normalized TEA-sensitive Kv3 current density versus age. Current density is obtained by dividing peak currents **(C)** by the capacitance of clamped membrane area **(B)**.

Inactivating TEA sensitive currents, presumed to be Kv3.3 currents, showed a significant increase with age (*n* = 14; P7–P17; Kendall τ: 0.43; *p* < 0.005; **Figure 3C**) but looking closer the increase was confined to the younger cells, saturating at p11 (Kendall τ = 0.57, *p *< 0.005). From p12 the current slightly dropped and had no significant correlation with age (Kendall τ = -0.23, *p* = 0.43). These results are not surprising: the passive electronic structure of PCs enlarges around p11 (Roth and Hausser, 2001; Sacco and Tempia, 2002) and voltage transients applied to the soma will cause currents that get filtered due to the cable properties of the extensive dendritic tree (Roth and Hausser, 2001), making it impossible to record dendritic currents via the somatic patch clamp pipette.

However, the postnatal development of the PC is not confined to a growing dendritic tree, but shows a change in structure as described in detail by McKay and Turner (2005) in the rat. Initially, the soma grows and quickly reaches its full size at around p6. These cells have multiple small dendrites on their soma, visible under differential interference contrast (DIC) video microscopy. Starting from p5, some cells retain the configuration with multiple dendrites, while others evolve to a more mature configuration characterized by a single primary stem dendrite. In the next phase the dendritic tree grows and becomes more complex, reaching its lateral boundaries at p15 (McKay and Turner, 2005). Afterward it grows toward the pial surface until p30 (Sadler and Berry, 1984). It is important to stress that our data was collected in mice which show an onset of the maturation 4 days earlier than in rats. We initially investigated the impact of morphological changes on the space clamp of PCs by measuring the capacitance by two sinusoidal waves of 50 and 500 Hz.

Purkinje Cells up to p9 were still compact and allowed an adequate space clamp as the 50 and 500 Hz capacitance measurements showed almost no difference (**Figure 3A**, filed and unfilled circles; Sacco and Tempia, 2002). Starting from p9 the capacitance measurement with the 500 Hz wave was quite distinct from the 50 Hz reflecting the massive expansion of the dendrite that filtered the fast sinus wave (**Figure 3A**; Spruston et al., 1993; Sacco and Tempia, 2002). Only at p11 the 500 Hz stimulus recorded an increased capacitance, showing that an immature dendrite can contribute to the recorded currents even for very fast currents.

While this method demonstrates the appearance of space clamp problems at p11, it cannot be used to normalize the Kv3.3b currents. Therefore, we estimated the clamped membrane area for each cell by integrating the capacitive transient over the same period during which Kv3 currents reach their peak amplitude. The capacitance of the clamped membrane area of growing PCs increased significantly until p12 (**Figure 3B**; Kendall τ = 0.70, *p* < 0.001), but became steady afterward (Kendall τ = 0.25, *p* = 0.43). The biggest membrane area that could get clamped occurred at p11. Coincidentally, these cells also showed the largest recorded peak current (**Figure 3C**).

The current density was calculated as the peak current divided by the capacitance of the clamped area of the cell and is named the normalized current density. This normalized current density had no correlation with age (Kendall τ: 0.06; *p* = 0.73; **Figure 3D**). The normalization eliminated the peak at p11 but increased the variability of the measurements with a CV of 45%. Note that we measured currents only in soma and proximal dendrite, we cannot exclude that Kv3.3 channels have a different current density in the dendrite compared to the soma, which might skew the observed CV.

### REGULATION OF Kv3.3 NORMALIZED CURRENT DENSITY

To test whether the phenotype was influenced by the level of transcription, we correlated the Kv3.3 normalized current density with the Kv3.3b mRNA level on a single cell basis, as described previously (Veys et al., 2012).

**FIGURE 4:**
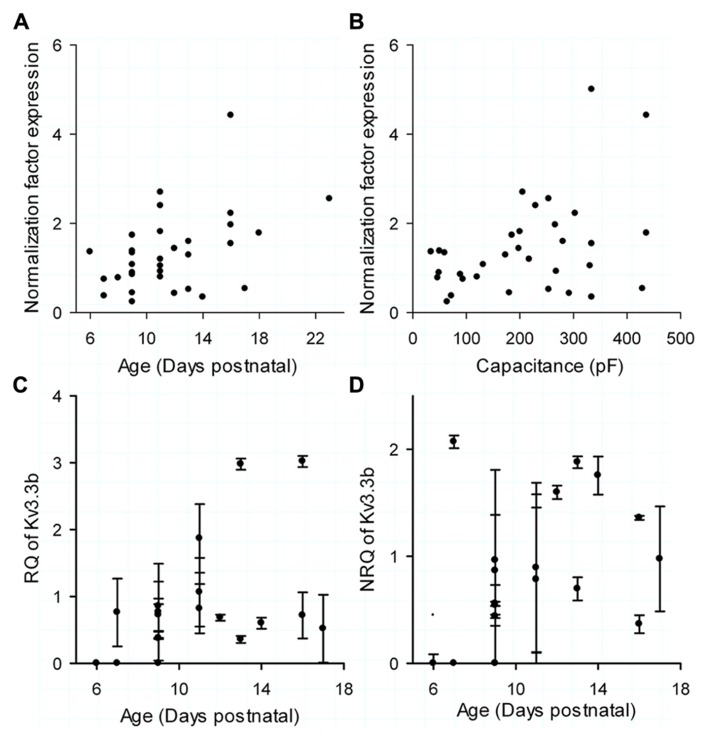
**Real time PCR data. (A)** Normalization factor (NF) of each cell, calculated as the average expression of the two housekeeping genes compared to postnatal age. **(B)** NF of each cell compared to total cell capacitance (measured by 500 Hz sinus wave). **(C)** Relative quantity (RQ) of Kv3.3b versus postnatal age. **(D)** Normalized relative quantity (NRQ) of Kv3.3b of each cell, calculated by dividing RQ **(C)** by NF **(A,B)** versus postnatal age. The error bars in **(C,D)** were determined through calculating the error propagation of the normalized or non-normalized relative quantity of the Kv3.3b real time PCR assay.

The normalization factor (NF) for the mRNA level, representing the combined expression of the housekeeping genes GAPD and calbindin, had no correlation with age (Kendall τ = 0.21, *p* = 0.075; **Figure 4A**) or cell size (**Figure 4B**; Kendall τ = 0.16, *p *= 0.18), which is a basic criterion when choosing housekeeping genes (Vandesompele et al., 2002). The relative expression (RQ; **Figure 4C**; linear regression with *R*^2^ = 0.21 with *p* > 0.05 and correlation with *R* = 0.45 and *p* > 0.05) and the normalized expression (NRQ; **Figure 4D**; linear regression with *R*^2^ = 0.19 with *p* > 0.05 and correlation with *R* = 0.34 and *p *> 0.05) of Kv3.3b showed no significant correlation or linear regression with age, which was consistent with the normalized current data (**Figure 3D**). The data represents 19 cells of which both real time PCR data and electrophysiology were available. We have previously shown that normalizing the data to housekeeping genes was vital to reduce variability arising from the extraction and all the following molecular reactions (Veys et al., 2012). As reported previously, normalization of our data decreased its CV from 102% for the not-normalized data to 66% for the normalized data.

**FIGURE 5:**
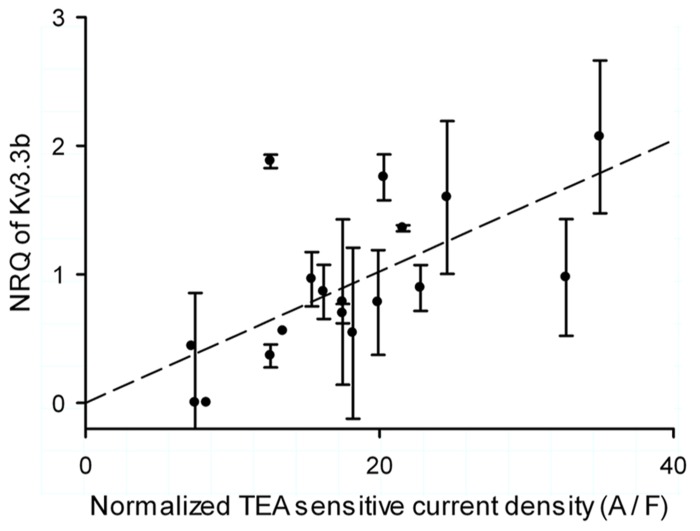
**Single cell correlation between current density and expression of Kv3.3b.** Normalized inactivating TEA sensitive Kv3.3 current density (normalized by capacitance of the clamped area of the cell) versus normalized relative quantity (NRQ) of Kv3.3b of each cell. The error bars were determined through calculating the error propagation of the normalized relative quantity of the Kv3.3b real time PCR assay.

Although neither normalized current density nor NRQ were correlated with age, they shared a very high coefficient of variation raising the question if Kv3.3b current density was regulated at level of its mRNA. This was indeed the case for the data set of 19 cells: the Kv3.3 normalized current density was significantly correlated with the normalized expression of Kv3.3b mRNA (**Figure 5**, Kendall τ with 0.34; *p* = 0.041 and Pearson correlation with *R* = 0.622 and *p* < 0.01).

### VARIABILITY IN Kv3.3b EXPRESSION AND THE SHAPE OF THE CS

**FIGURE 6:**
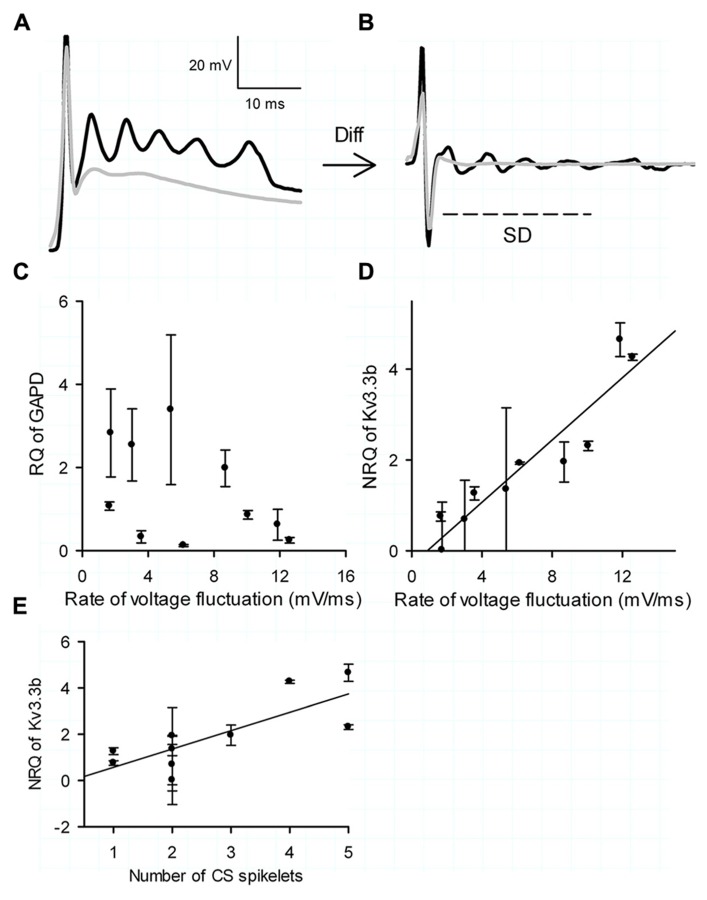
**Kv3.3b and the complex spike. (A)** Examples of current clamp measurements of a CS with five spikeletes (black trace) and one with a single spikelet (gray trace). **(B)** Differentiation of examples shown in **(A,B)**. Rate of voltage fluctuation of the CS represented by the SD of the first 10 ms after the initial full action (arced line). **(C)** Relative expression of the housekeeping gene GAPD versus rate of voltage fluctuation. **(D)** Normalized relative expression (NRQ) of Kv3.3b versus rate of voltage fluctuation. **(E)** NRQ of Kv3.3d versus number of CS spikelets. The error bars in **(C,D)** were determined through calculating the error propagation of the normalized or non-normalized relative quantity of the Kv3.3b real time PCR assay.

It was already established by Zagha et al. (2008) that Kv3.3b is the sole channel repolarizing the spikelets in the CS (referring to their **Figure 3**) and that the CSs show intrinsic differences in shape and number of spikelets (referring to their **Figure 5**). We measured the rate of voltage fluctuation of the spikelets in the CS using their protocol, i.e., as the standard deviation of the first derivative in the 10 ms windows after the first spike. (**Figures 6A,B**). These experiments were done in mice form age 18 to 24, similar to Zagha et al. (2008). After extraction of the mRNA from the same cells, one housekeeping gene was used (GAPD) as a control, which showed no significant correlation with the rate of voltage fluctuation of the CS (**Figure 6C**; Pearson correlation with *R = *-0.34 and *p* = 0.35). Conversely the NRQ of Kv3.3b showed a very significant linear correlation with the voltage fluctuation of the spikelets (**Figure 6D**; Pearson correlation with *R* = 0.94 and *p *< 0.0001). The number of spikelets in the CS also showed a significant correlation with the normalized quantity of Kv3.3b (**Figure 6E**; Pearson correlation with *R* = 0.78 and *p *< 0.01).

The CV for the NRQ data in **Figure 5** was 78%. By using the Pfaffl (2001) method for normalizing the real time data we could calculate the SD for every assay, which were combined to a pooled variance of 48%. This represented the experimental noise arising from the real time PCR, mRNA extraction, and amplification. Subtracting this experimental error from the CV for the NRQ data yields a CV of 30% representing the actual variation of Kv3.3b expression in the PC.

## DISCUSSION

The CS is considered to be an all-or-nothing phenomenon, but it clearly shows intrinsic differences in the number and shape of spikelets when elicited by a sole square wave pulse stimulation of the climbing fiber (Eccles et al., 1966; Zagha et al., 2008). Knock-out and blocking experiments have shown that somatic Kv3.3b is the most important channel responsible for the repolarization of these spikelets (Zagha et al., 2008) and have therefore the possibility of regulating intrinsic differences seen in the CS.

### DEVELOPMENTAL REGULATION OF Kv3.3b EXPRESSION

Kv3.3b has been proposed to be under developmental control (McKay and Turner, 2005) based on previous observations of Goldman-Wohl et al. (1994), whom identified the alternatively spliced form Kv3.3b in the PC (Goldman-Wohl et al., 1994). These experiments used immunochemistry with antisense RNA probes labeled with DIG and detected Kv3.3b expression starting between p8 and p10 in mice. However, our experiments used a more sensitive approach with single-cell real-time PCR, a technique which has been shown to be sensitive enough to correlate single cell mRNA levels with patch clamp data in both expression systems and cultured neurons (Veys et al., 2012). Using this optimized technique we could detect Kv3.3b from p6. At this age the soma of the mouse PC reached its mature size, and growth of the neuron is restricted to the increase in size and branching complexity of the dendritic tree (Sadler and Berry, 1984; Scelfo et al., 2003; McKay and Turner, 2005). Therefore, the techniques used by Goldman-Wohl et al. (1994) may not have detected Kv3.3b in a fully-grown soma and the reported developmental up regulation could have represented dendritic growth as Kv3.3b is expressed throughout the entire cell (Rashid et al., 2001; McKay and Turner, 2004; Zagha et al., 2008). In contrast, the spikelets in the CS are repolarized solely by somatic Kv3.3b channels (Zagha et al., 2008) with no influence of dendritic expressed channels (Davie et al., 2008).

Our results show a significant increase of Kv3.3b current during maturation up to p11, similarly as shown by Goldman-Wohl et al. (1994). We could not detect an increase in total current in older and larger cells due to limitations of somatic single electrode voltage clamp, which was necessary for mRNA extraction. Normalizing the current to the clamped area showed that the channel density is stable during the maturation of the PC.

Although Kv3.3b normalized current density seemed stable during early PC development, the data showed large cell-to-cell differences, which was not really surprising. Both modeling and experimental work have shown that neurons can achieve the same amount of net inward and outward current by combining different sets of voltage-gated channels (Swensen and Bean, 2005; Achard and De Schutter, 2006; Baumgardt et al., 2007; Schulz et al., 2007). However, subtle changes in one of the currents can have dramatic effects on the activity of the neurons, raising the question why PCs show these variations and how they are regulated?

To answer the second question we quantified the expression of Kv3.3b by real time PCR in the same cells where we recorded the currents as described recently (Veys et al., 2012). The normalized relative quantity of Kv3.3b mRNA showed no correlation with age but showed a high cell-to-cell variability, similar to that of the normalized Kv3.3 current density.

mRNA and proteins levels are directly linked by translation except when post-translational, translational, or protein degradation mechanisms alter their relation which is believed to be the case for two thirds of the genes (Vogel and Marcotte, 2012). As the normalized Kv3.3b current density correlated with the normalized relative expression (**Figure 5**, Pearson correlation with *R* = 0.622 and *p* < 0.01), it is most likely regulated by the size of the Kv3.3b mRNA pool without a significant contribution of processes beyond transcription. Experimental limitations both with voltage clamp control and quantitative PCR may have influenced our conclusions, but overall our findings are similar to experimental data shown in crab stomatogastric lateral pyloric neurons where a tight correlation between mRNA and their respective potassium currents was found (Schulz et al., 2007).

We used two housekeeping genes to eliminate any correlation of the normalizing factor of the expression with the size of the cell, which varied a lot in the maturing PC. The amount of cDNA available even after pre-amplification was still limited and the extra assay done in twofold substantially lowered the success rate to 36% compared to 52% in the CS experiments where only one housekeeping gene was used, similarly as described in Veys et al. (2012).

### Kv3.3b AND THE COMPLEX SPIKE

Several studies have reported that CSs in the soma show large variation in waveforms from cell to cell and sometimes also within the cell (Llinas and Sugimori, 1980; Hansel and Linden, 2000; Davie et al., 2008; Mathy et al., 2009). In a computational study we recently reported that stochastic activation of the calcium-activated mslo channel is the main cause of the large spatiotemporal variability of dendritic CS waveforms (Anwar et al., 2013), but variability of channel expression was not explored. Here we demonstrate a strong effect of transcriptional variation causing variable channel expression on the somatic shape of the CS. While our studies address different causes of CS waveform variability, the functional significance of this variability remains unresolved (Hong and De Schutter, 2008, p. 329; Mathy et al., 2009).

As Kv3.3 has been reported as the most important channel repolarizing spikelets in the CS the observed cell-to-cell differences in Kv3.3b mRNA and normalized current density could have an impact on the shape of the CS. We showed a strong and significant correlation between the normalized relative quantity of Kv3.3b mRNA and both the number of spikelets in the CS and their rate of voltage fluctuation. The intrinsic CS shape was thereby directly related to the Kv3.3b mRNA pool, which at this age shows large variability directly affecting Kv3.3 current (**Figure 4**). Note that Zagha et al. (2008) reported that only somatic Kv3.3 channels are necessary for the repolarization of the spikelets and not dendritic channels. Our measurement of Kv3.3b mRNA from the whole cell does not allow discriminating between dendritic or somatic targeting of the final protein. Currently, it is unknown if kv3.3 channel density is strongly different between the soma and the dendrite and how this would affect our conclusion.

The ionic mechanism of the CS has its roots in the interaction between the Kv3.3 channels and resurgent sodium channels (Akemann and Knopfel, 2006; Zagha et al., 2008). These channels show a state of open block allowing recovery without a state of inactivation (Raman and Bean, 1997), which is not possible in the CS because of the calcium plateau that prevents deep spike repolarization. The kinetic properties of the Kv3.3 channels allow for fast repolarization and relief of open block as show in the context of high-frequency firing in PCs (Raman and Bean, 1999; Swensen and Bean, 2003; Akemann and Knopfel, 2006) as well as in the CS (Zagha et al., 2008). Dynamic clamp experiments using the CS waveform as a voltage command showed (Zagha et al., 2008; **Figure 6**) that 75–90% of the resurgent sodium channels are blocked and all non-resurgent sodium channels are inactivated after the first spike (Zagha et al., 2008). Experiments with TEA, blocking Kv3.3 channels, showed that an equal amount of channels accumulated in open block and inactivated state after the initial spike compared to the control situation without TEA (Zagha et al., 2008). Only in experiments without TEA a rapid recovery of the resurgent sodium channels during spikelets was observed (Zagha et al., 2008). This data convinced us that possible variation in the amount of resurgent sodium channels would be of minor importance in the shaping of the CS compared to variation in the amount Kv3.3 channels.

The question remains that if this variation is controlled at the level of the mRNA, whether this is merely transcriptional noise or part of homeostatic processes (Marder and Goaillard, 2006).

Studies on transcriptional noise have reported CV values ranging from 10 to 65% in bacteria, 8 to 42% in yeast (Ozbudak et al., 2002; Raser and O’Shea, 2004) and 10–37% in mammalian macrophages (Ramsey et al., 2006). Currently, there is no data on phenotypic variance of channel proteins or mRNA in neurons, which is not surprising as it is very challenging to measure since the variance is dependent on the promoter (Raser and O’Shea, 2004) and therefore possibly different for every channel. The CV, after adjusting for experimental errors, was 30% for our CS data, which is of the same magnitude as reported in other systems, suggesting that the variation of Kv3.3b in the PC could be due to transcriptional noise. Although, a definite conclusion can only be made after future experiments on the transcriptional noise of the Kv3.3b promoter in PCs.

Unfortunately, it was not possible with current methodology to explore possible relations between markers for compartmentalization, such as zebrin II (Apps and Hawkes, 2009) and variability in Kv3.3 expression. Other receptors like Gaba_B2_ (Chung et al., 2008) and mGLu_R1_ (Wang et al., 2011) have been show to co-localize with zebrin II positive PCs. Currently, there is no such data available for Kv3.3 and remains to be explored. However both Gaba_B2_ and mGLu_R1_ receptors show an all or nothing expression pattern which is very distinct from the graded Kv3.3b expression reported here, making it unlikely that similar mechanism are in play.

In conclusion our results clearly show that the PC do not tightly control the normalized Kv3.3 density during maturation by transcription and this variability of expression influences the shape of the CS as it is the most important channel repolarizing the spikelets.

## Conflict of Interest Statement

The authors declare that the research was conducted in the absence of any commercial or financial relationships that could be construed as a potential conflict of interest.
